# Temporal Regulation of the Transformasome and Competence Development in *Streptococcus suis*

**DOI:** 10.3389/fmicb.2016.01922

**Published:** 2016-12-20

**Authors:** Edoardo Zaccaria, Michiel Wels, Peter van Baarlen, Jerry M. Wells

**Affiliations:** ^1^Host-Microbe Interactomics, Animal Sciences, Wageningen UniversityWageningen, Netherlands; ^2^NIZO Food Research B. V.Ede, Netherlands

**Keywords:** *Streptococcus suis*, competence, DNA transformation, sigma factor X, fratricin, pillus, bacteriocins

## Abstract

In *S. suis* the ComX-inducing peptide (XIP) pheromone regulates ComR-dependent transcriptional activation of *comX* (or *sigX*) the regulator of the late competence regulon. The aims of this study were to identify the ComR-regulated genes and in *S. suis* using genome-wide transcriptomics and identify their function based on orthology and the construction of specific knockout mutants. The ComX regulon we identified, includes all homologs of the “transformasome” a type 4-like pilus DNA binding and transport apparatus identified in *Streptococcus pneumoniae, Streptococcus mutans*, and *Streptococcus thermophilus*. A conserved CIN-box (YTACGAAYW), predicted to be bound by ComX, was found in the promoters of operons encoding genes involved in expression of the transformasome. Mutants lacking the major pilin gene *comYC* were not transformable demonstrating that the DNA uptake pilus is indeed required for competence development in *S. suis*. Competence was a transient state with the *comX* regulon shut down after ~15 min even when transcription of *comX* had not returned to basal levels, indicating other mechanisms control the exit from competence. The ComX regulon also included genes involved in DNA repair including *cinA* which we showed to be required for high efficiency transformation. In contrast to *S. pneumoniae* and *S. mutans* the ComX regulon of *S. suis* did not include *endA* which converts the transforming DNA into ssDNA, or *ssbA*, which protects the transforming ssDNA from degradation. EndA appeared to be essential in *S. suis* so we could not generate mutants and confirm its role in DNA transformation. Finally, we identified a putative homolog of fratricin, and a putative bacteriocin gene cluster, that were also part of the CIN-box regulon and thus may play a role in DNA release from non-competent cells, enabling gene transfer between *S. suis* pherotypes or *S. suis* and other species. *S. suis* mutants of *oppA*, the binding subunit of the general oligopeptide transporter were not transformable, suggesting that it is required for the import of XIP.

## Introduction

The process of natural competence for DNA transformation in specific habitats or “natural competence” has been established as an important mechanism impacting on bacterial evolution and speciation. A nutritional benefit of natural competence has also been proposed through the provision of DNA nucleotides generated by degradation of one DNA strand during transport into the cytoplasm. The genes associated with natural competence are widely distributed throughout the bacterial kingdom, although experimental evidence for natural competence is limited to only a few genera.

Most streptococcal species belonging to the *Streptococcus mutans, Streptococcus thermophilus*, and *Streptococcus gordonii* phylogenetic groups possess conserved genetic components of the competence machinery (Johnston et al., 2014), and natural competence has been experimentally demonstrated in around 16 species of *Streptococcus* (Håvarstein et al., 1995; Fontaine et al., 2010; Mashburn-Warren et al., 2010; Morrison et al., 2013; Zaccaria et al., 2014). In streptococci competence is induced by an alternative sigma factor, ComX or SigX, which regulates expression of the late competence genes encoding functions in DNA uptake and recombination. Two main types of pheromone regulatory systems control the proximal regulatory switch for *comX* expression. The first is exemplified by *Streptococcus pneumoniae*, which uses a two-component system to sense and respond to a competence stimulating peptide (CSP) by inducing ComX. This alternative sigma factor controls the late competence regulon via interaction with the Com box motifs (also known as CIN-Box motifs) in promoter DNA sequences and interaction with RNA polymerase. The late competence regulon includes operons encoding genes for assembly of the type 4-like pilus, a DNA uptake system (Laurenceau et al., 2013) generating a single stranded DNA (ssDNA) molecule, and DNA recombination and repair enzymes that promote formation of the recombination synapse, heteroduplex formation and strand exchange between homologous DNAs (Campbell et al., 1998; Luo and Morrison, 2003; Peterson et al., 2004).

A second pheromone regulatory system for induction of competence was discovered in *S. mutans, S. thermophilus*, and *S. pyogenes*. In these species, ComR, an Rgg family transcriptional activator, positively regulates expression of *comX* and *comS*, through allosteric interaction with a processed form of the pheromone encoded by *comS* (Mashburn-Warren et al., 2010, 2012; Gardan et al., 2013; Zaccaria et al., 2014). The mature pheromone peptide induces competence from outside the bacteria but its mechanism of export is unknown. In *S. mutans* and *S. thermophilus*, the import of the mature pheromone is dependent on Opp, a general peptide transporter (Gardan et al., 2009; Guo et al., 2014).

The Opp (or Ami) peptide transport system is essential for competence development of *S. mutans* and *S. thermophilus* (Gardan et al., 2009, 2013; Mashburn-Warren et al., 2010; Fleuchot et al., 2011), and it appears to be responsible for the internalization of the XIP. The Opp transporters consist of two transmembrane hydrophobic pore-forming domains (OppB and OppC) and two ATP-binding proteins (OppD and OppF), that hydrolyse ATP to provide the energy required for peptide transport (Higgins, 2001). In addition to these conserved proteins, the Opp operon encodes a ligand-binding protein (OppA) that is responsible for recognizing and binding extracellular peptides, thus conferring specificity to the transport system.

In streptococci, competence is a transient physiological bacterial state (Seaton et al., 2011, 2015; Desai et al., 2012; Federle and Morrison, 2012; Guo et al., 2014) and the mechanisms mediating shut-down have only been partially elucidated in some species (Boutry et al., 2012; Tian et al., 2013; Weng et al., 2013; Dong et al., 2014; Wahl et al., 2014). In *S. pneumoniae*, which possess the ComCDE competence regulatory system, the exit from competence is regulated by multiple ComX dependent- and independent mechanisms (Chastanet et al., 2001; Bergé et al., 2003; Mortier-Barrière et al., 2007; Piotrowski et al., 2009; Martin et al., 2013; Mirouze et al., 2013; Weng et al., 2013) In *S. mutans* and *S. thermophilus* which both utilize the ComRS system to regulate competence development, MecA negatively regulates competence development by targeting the ClpC-ClpP protease activity to ComX (Boutry et al., 2012; Tian et al., 2013). Moreover, *in vitro* degradation of ComX by ClpC-ClpP was shown to be strictly dependent on MecA (Wahl et al., 2014).

In some streptococci including *S. pneumoniae* ComX regulates secondary processes including expression of stress response pathways and fratricin, a cell wall hydrolase which provides a predatory mechanism to lyse non-competent pneumococci and acquire DNA (Kausmally et al., 2005; Håvarstein et al., 2006; Claverys et al., 2007). Conservation of this predatory mechanism has been proposed in other streptococci based on gene homologies and the presence of CIN-boxes in promoter regions (Berg et al., 2012). Recently, in *S. mutans* a bacteriocin-like molecule was identified that is induced by its competence-inducing peptide, causing autolysis in part of the population (Perry et al., 2009; Lemme et al., 2011).

We recently identified a pheromone-induced mechanism of competence in *Streptococcus suis*, an important pig pathogen and zoonotic agent of human meningitis (Zaccaria et al., 2014). The competence system of *S. suis* appears to be similar to the ComRS-driven mechanism that has been discovered in *S. mutans, S. thermophilus*, and *S. pyogenes*, although *S. suis* belongs to a different phylogenetic group (Zaccaria et al., 2014).

A time-series transcriptome study of competence development has been previously reported for streptococcal species using a two-component system to regulate peptide-induced competence development (Dagkessamanskaia et al., 2004; Vickerman et al., 2007) but as far as we are aware similar studies have not been performed for a streptococcal species harboring a ComRS system as the proximal switch. The aims of this study were to identify the ComR-regulated genes and in *S. suis* using genome-wide transcriptomics and identify their function based on orthology and the construction of specific knockout mutants. At three biologically relevant times after pheromone induction of competence (Zaccaria et al., 2014), *S. suis* RNA was extracted and hybridized to commercially available whole-genome microarrays. We found that induction and repression of major DNA repair and RNA metabolic genes occurred within 5 and 15 min, indicating that uptake, processing and incorporation of exogenous DNA into the *S. suis* genome occurs effectively within 15–30 min. Our data were used to predict the *S. suis* transformasome by orthology and pinpoint processes that are both crucial to genomic integrity and gene transfer. These processes are therefore not only relevant from a fundamental biological viewpoint but could also be targets of future antimicrobials.

## Materials and methods

### Bacterial strains and culture conditions

The *S. suis* strains used in the present study are listed in Table 1. *S. suis* strain S10 is a virulent isolate from an infected pig, and its genome is 99% identical to the genome of *S. suis* 2 strain P1/7 (de Greeff et al., 2011), a sequenced reference strain of which the genome had been annotated previously (Holden et al., 2009). *S. suis* was grown at 37°C at 5% atmospheric CO_2_ in Todd Hewitt Broth (THB, Thermo Scientific, Oxoid) or on THB plates containing 1.2% of agar (BD). When required the medium was supplemented with spectinomycin (Invitrogen) and/or chloramphenicol (Sigma) at a concentration of 100 and 5 μg/ml, respectively. Insertional deletion mutants of the genes *cinA, oppA*, and *comYC* were constructed in *S. suis* strain S10 by Gene Splicing Overlap Extension PCR (SOE-PCR) and allelic replacement as previously described (Zaccaria et al., 2014). The primers used for SOE-PCR are shown in Table 1. Successful deletion of the genes was verified by colony PCR using primer combinations based on DNA sequences of the inserted DNA and proximal chromosomal DNA (Table 1) and verified by sequencing of the amplicons. Growth phase was determined by measuring optical density at 600 nm (OD_600*nm*_) using a SpectraMax M5 reader (Molecular Devices LLC).

**Table 1:** *****S. suis*** strains and primers used**.

**Strain**	**Relevant characteristics**	**Source of reference**
*S. suis* S10	Wild-type; reference strain	Vecht et al., 1996; Zaccaria et al., 2016a
*S. suis* ΔcinA	S10 ΔcinA	This study
*S. suis* ΔoppA	S10 ΔoppA	This study
*S. suis* ΔcomYC	S10 ΔcomYC	This study
**Primers**	**Nucleotide sequence**	**Purpose**
CinA 1F	TGCGGCCATGACAGATAGCG	Creation of *cinA* deletion fragment
CinA 1R	CTTGCCAGTCACGTTACGTTTCCGTCCCAACGGCGATTAG	Creation of *cinA* deletion fragment
CinA 2F	CTAATCGCCGTTGGGACGGAAACGTAACGTGACTGGCAAG	Creation of *cinA* deletion fragment
CinA 2R	GTCCTCTGTTGATTCCGGTTTCGGTACCCTATGCAAGGGTTT	Creation of *cinA* deletion fragment
CinA 3F	CCTTGCATAGGGTACCGAAACCGGAATCAACAGAGGACAAC	Creation of *cinA* deletion fragment
CinA 3R	TCTTTCTGGGCTTGAGCTACTG	Creation of *cinA* deletion fragment
CinA ctrl F	GGAGTTTCTATGTCCCGTTGTG	Control of ΔcinA
CinA ctrl R	GTACAAGGGCTGCAACCGAGTC	Control of ΔcinA
OppA 1F	CGGAAACCGACGTGTAAATC	Creation of *oppA* deletion fragment
OppA 1R	CTTGCCAGTCACGTTACGTTTCGGGTACAGGTCTTGCTTATG	Creation of *oppA* deletion fragment
OppA 2F	TAAGCAAGACCTGTACCCGAAACGTAACGTGACTGGCAAGAG	Creation of *oppA* deletion fragment
OppA 2R	GTTCTTGCAGCATGTGGTTCTCGGTACCCTATGCAAGGGTTTA	Creation of *oppA* deletion fragment
OppA 3F	AACCCTTGCATAGGGTACCGAGAACCACATGCTGCAAGAACAGA	Creation of *oppA* deletion fragment
OppA 3R	CACGAATGCAGCTTCGCTACC	Creation of *oppA* deletion fragment
OppA ctrl F	GTTATGCAAGCCCATGATGGTC	Control of ΔoppA
OppA ctrl R	AGGCGTTTAGCGAGGTAATGTC	Control of ΔoppA
ComYC 1F	ACCTACCTGACGGCCTATTACG	Creation of *comYC* deletion fragment
ComYC 1R	TCTCTTGCCAGTCACGTTACGTTCCACCAGAGTGAACCCTTT	Creation of *comYC* deletion fragment
ComYC 2F	AAGGGTTCACTCTGGTGGAACGTAACGTGACTGGCAAGAGAT	Creation of *comYC* deletion fragment
ComYC 2R	CCTTTCGCCTGCAAATCTGCTCGGTACCCTATGCAAGGGTTTA	Creation of *comYC* deletion fragment
ComYC 3F	ACCCTTGCATAGGGTACCGAGCAGATTTGCAGGCGAAAGGTT	Creation of *comYC* deletion fragment
ComYC 3R	CTGGACAGCCATCTGTGCTAAG	Creation of *comYC* deletion fragment
ComYC ctrl F	GATTGAGGTGGCGACCTATCCG	Control of ΔcomYC
ComYC ctrl R	AGAGGCTACCTGACAGAATGAC	Control of ΔcomYC
Spec F	ACCGTGGAATCATCCTCCCAAAC	Control of all mutants
Spec R	CCACTGCATTTCCCGCAATATC	Control of all mutants

### RNA extraction

RNA was isolated and purified from *S. suis* cultures at different times after induction of competence. Briefly *S. suis* S10 was grown to OD_600nm_ 0.04. Thirty-five mL of culture was collected and transforming DNA (pNZ8048, 350 μg) in EB buffer (10 mM Tris-Cl, pH 8.5) was added to the bacteria together with synthetic XIP (GNWGTWVEE) at a final concentration of 250 μM. At 5, 15, and 45 min after the addition of XIP, 10 mL aliquots of the cultures were centrifuged for 2 min at 8000 g at RT and the bacterial pellets resuspended in 2.5 mL PBS plus 5 mL RNAprotect buffer (Qiagen). After 5 min incubation the bacterial suspension was centrifuged, the supernatant aspirated, and bacterial pellet immediately frozen in liquid nitrogen. The frozen pellet was dissolved in 110 μL of TE containing proteinase K and lysozyme (1.25 and 15 μg/ml, respectively) and incubated for 10 min at room temperature with vortex mixing every 2 min. Then 700 μL of RLT buffer (Promega) containing 7 μL of freshly added β-mercaptoethanol was added and the bacteria disrupted using a FastPrep-24 (MP Biomedicals, Solon, OH) for 20 s at 6.0 m/s. Total RNA was purified using the RNeasy Mini Kit (Qiagen). The quality and the concentration of RNA were determined using the Experion System (Bio-Rad) and measurement of the A260/A280 ratio (NanoDrop 8000 UV-Vis Spectrophotometer). Complementary DNA (cDNA) was synthesized using the SuperScript III Reverse Transcriptase kit (Invitrogen) using aminoallyl-dUTPs in place of UTP and purified with the Illustra CyScribe GFX Purification Kit (GE Healthcare). The cDNA was labeled with CyDye Post-Labeling Reactive Dye Pack (GE Healthcare) using the manufacturer's recommended protocol. RNA was also purified from control cultures to which no XIP or no transforming DNA was added using the above method.

### Microarray transcriptome analysis

An *S. suis* oligoarray (8 × 15 K) containing *in situ* synthesized 60-mers was produced by Agilent Technologies (Santa Clara, USA), based on the genome sequence of *S. suis* P1/7 (Holden et al., 2009). A total of 7651 unique 60-mers having a theoretical melting temperature of ~81°C and representing 1960 ORFs were selected as described (Saulnier et al., 2011). Genes were represented by 4 (91%), 3 (4%), 2 (2%), or 1 probe(s) (3%). Twenty-five putative genes were not represented on the array because no unique probe satisfying the selection criteria could be selected. Co-hybridization with labeled cDNA probes was performed on these oligonucleotide arrays at 42°C for 16 h in hybridization chambers (Slidehyb#1, Ambion, Austin, USA). The data were normalized using Lowess normalization (Yang et al., 2002) as available in MicroPrep (van Hijum et al., 2003) and corrected for inter-slide differences on the basis of total signal intensity per slide using Postprep (van Hijum et al., 2003). Significance of differential gene expression was based on false discovery rate (FDR) values lower than 0.05. The data discussed in this publication have been deposited in NCBI's Gene Expression Omnibus and are accessible through GEO Series accession number GSE74507.

### Microarray data analysis

Within the dataset gene expression data with high standard deviation (>250) or very low expression values (i.e., not having at least four observations with absolute value higher than 20) or that were not altered by the induction of competence (maximal value minus minimal value of at least 200) were filtered out using Cluster 3.0. Further details on the software and settings can be found in the online handbook (http://bonsai.hgc.jp/~mdehoon/software/cluster/cluster3.pdf). Heatmaps were generated by the MultiExperimental Viewer (MeV) program (http://www.tm4.org/mev.html) (Saeed et al., 2006).

### Transformation experiments

*S. suis* strains were grown overnight in THB broth in an incubator 37°C with 5% CO_2_. The overnight culture was then diluted 1:40 into pre-warmed THB broth, and grown at 37°C without shaking. When the culture reached an OD_600nm_ of ~0.04, aliquots of 100 μL were transferred to 1.5 mL Eppendorf Safe Lock Tubes™ and combined with transforming DNA (1.2 μg of pNZ8048) in EB buffer (10 mM Tris-Cl, pH 8.5) and 5 μl of XIP at a final concentration of 250 μM. After 2 h of incubation at 37°C in the presence of 5% CO_2_, the samples were diluted and plated onto THB agar plates containing antibiotic.

## Results and discussion

### The competence pheromone induces distinct clusters of differentially regulated genes at specific time-points

**Figure 1:**
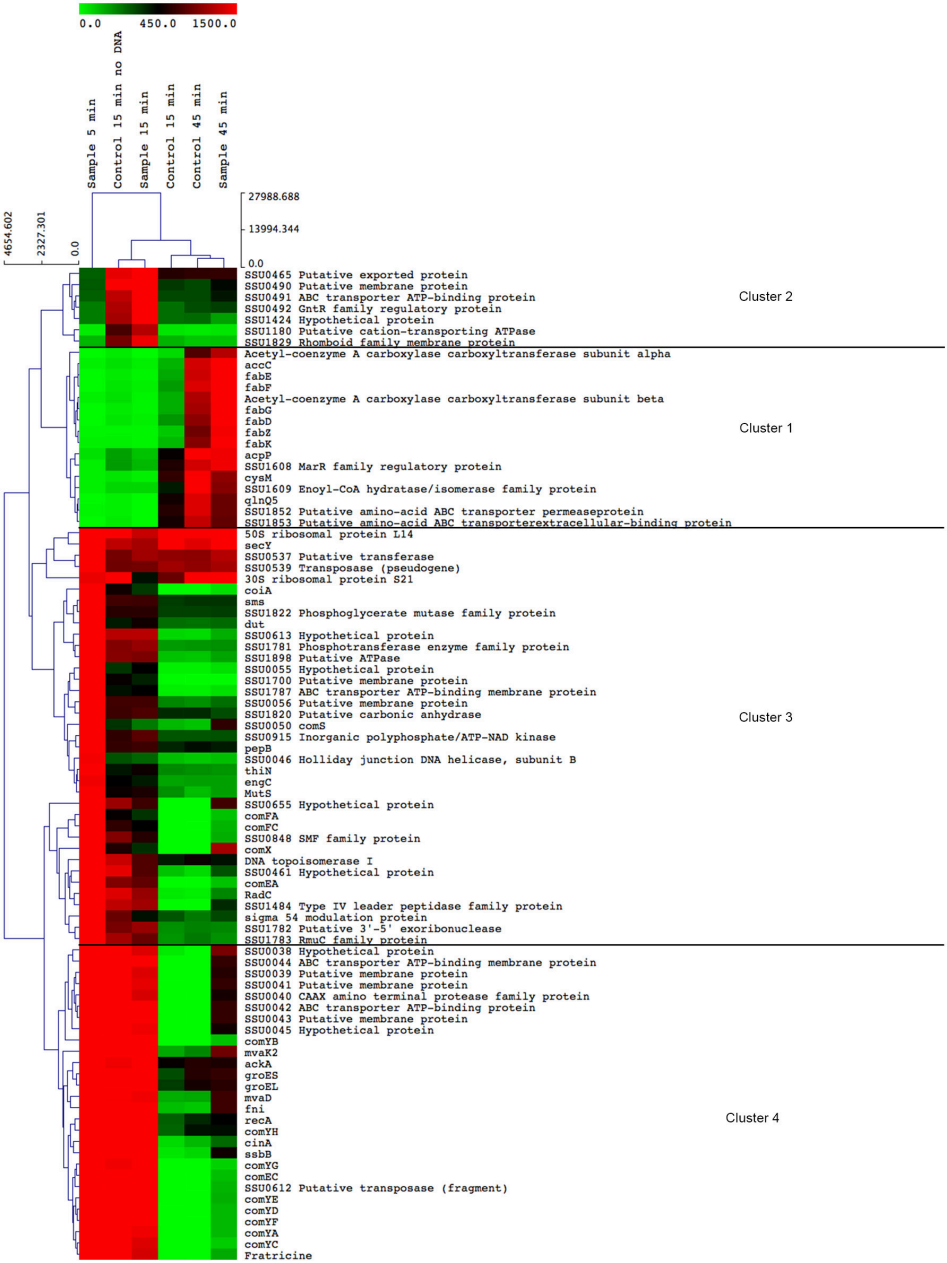
**Heatmap displaying the most differentially expressed genes in ***S. suis*** during competence**. Genes were filtered using Cluster 3.0 (See Methods) and were clustered using average linkage and Euclidian distance using MultiExperiment Viewer (MeV, see Methods). The color scale at the top depicts the normalized, unlogged expression values of the genes indicated on the right. The heatmap colors represent gene expression levels from the lowest value (zero, light green) to the highest level (~3000, bright red). To not lose resolution of intermediary expression levels, a highest cut-off value of 1500 was applied. The time at which bacterial samples were rapidly centrifuged and suspended in RNAprotect buffer is indicated above each column. “Control” indicates bacterial cultures to which no inducing peptide was added. “No DNA” indicates that no transforming DNA was added together with the competence inducing peptide.

Competence pheromone-induced transcriptional changes were identified by microarray analysis of RNA isolated from bacteria at 5, 15, and 45 min in the presence and absence of the competence pheromone and in the presence of exogenous DNA as previously described (Zaccaria et al., 2014). Additionally, the peptide pheromone was added without adding DNA, to identify possible effects of DNA addition. Five minutes after addition of the competence pheromone, 556 differentially expressed genes were up-regulated more than two-fold and 215 genes were down-regulated more than two-fold. At 15 min 148 and 185 differentially expressed genes were respectively up-regulated or down-regulated. At 45 min 140 and 48 were up- and downregulated, respectively. Genes that were not expressed or did not change expression at the multiple time points and controls were removed by filtering the data as described in Methods. Genes with altered expression were clustered according to their relative expression values at the different time points (Figure 1). Four major clusters were observed (Figure 1), the first of which (cluster 1) contains genes that were down-regulated upon induction of competence until 45 min post-addition of peptide (Figure 1). Cluster 1 contains 13 of the 14 fatty acid biosynthetic pathway genes and three genes involved in cell envelope metabolism. This reflects the finding that cell division and basal metabolic processes are halted during competence development (Zaccaria et al., 2016b) to avoid recombination of transforming DNA during DNA replication which is potentially dangerous to genome integrity. Cluster 2 contains genes of diverse functions including a cation-transporting ATPase, a putative peptidase and a predicted transcriptional regulator that were up-regulated only at 15 min. Cluster 3 genes were all highly (>4 fold) up-regulated at 5 min, after which their expression decreased until 45 min, when expression reached the same level as measured for the uninduced control samples. Cluster 3 contained 37 genes including *comX*, the sigma factor controlling the competence regulon. Of these 37 genes, 8 genes were annotated to be involved in DNA repair and recombination, and four genes were annotated as homologs of the multi-protein Type 4 pilus-like DNA uptake and transport apparatus recently described as the “transformasome” in *S. pneumoniae* (Laurenceau et al., 2013). Cluster 4 contains 28 genes that were highly expressed at 5 and 15 min and downregulated at 45 min including the *comYA*-*YH* operon encoding homologs of pneumococcal proteins forming the transformasome DNA uptake apparatus, genes encoding chaperones *groES and groEL*, a putative fratricin gene, three genes in the mevalonate pathway and an operon (SSU0038-SSU0045) of unknown function.

### The *S. suis* transformasome is regulated by ComX via a conserved CIN-box

**Figure 2:**
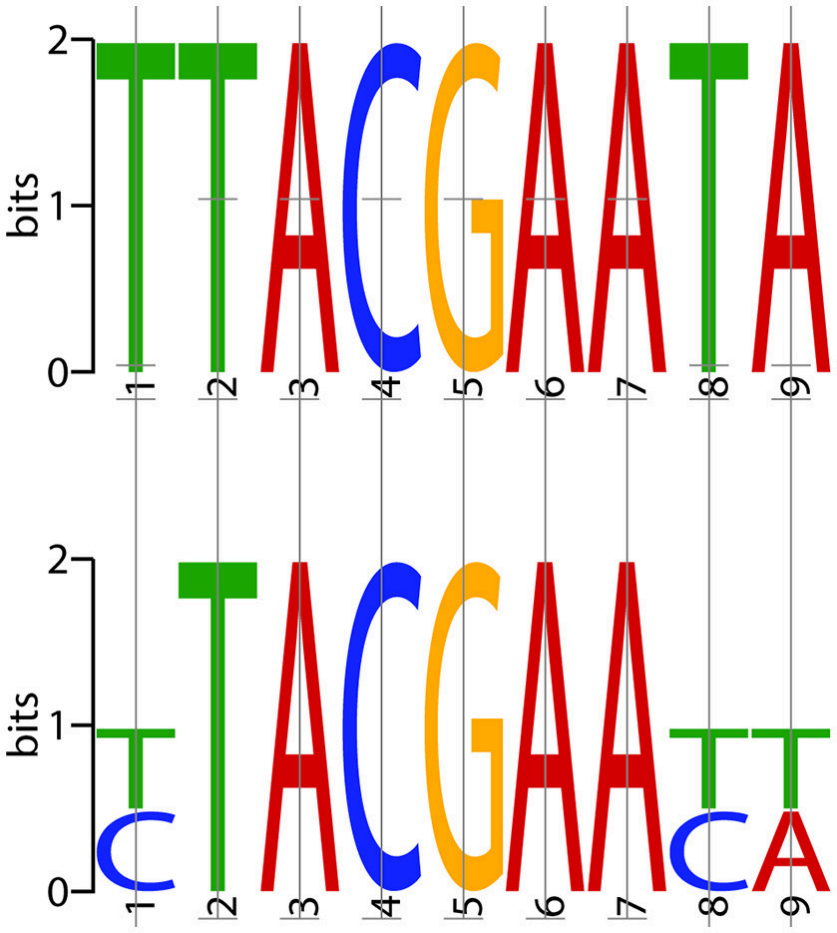
**Consensus CIN-box of ***S. pneumoniae*** and ***S. suis*****. The consensus CIN-box identified in *S. pneumoniae* (top) is compared to that identified for *S. suis* (bottom) using MEME with the FIMO plug in (http://meme-suite.org/doc/overview.html). The MEME motifs represent the probability of each possible DNA nucleotide appearing at each possible position in an occurrence of the motif. The possible letters are *A, C, G*, and *T*, adenine, cytosine, guanine, or thymine, covalently linked to a phosphodiester backbone. The height of the individual letters in a stack represents the probability of the letter at that position.

To identify the consensus motifs interacting with ComX in *S. suis* (i.e., CIN-box), promoters of genes or operons that were highly upregulated at 5 and 15 min in the presence of the competence peptide but in not the control samples at the same points were searched for consensus motifs using MEME (http://meme-suite.org/doc/overview.html). The conserved consensus 9 nt motif (YTACGAAYW) identified in *S. suis* is similar to the CIN-box of *S. pneumoniae* (Figure 2). In *S. suis* the CIN-box genes were present in nine operons, four of which were identified using the FIMO module of the MEME software suite (See Methods). The *S. suis* CIN-box genes encode homologs of all the known transformasome proteins in *S. pneumoniae, S. mutans*, and *S. thermophilus* (Table 2), showing its conservation across streptococcal species (Peterson et al., 2000, 2004; Vickerman et al., 2007).

**Table 2:** **Transformasome genes under ComX regulation with their homologs in ***S. mutans, S. pneumonia, and S. thermophilus*** and their (putative) function**.

**ComX regulated genes**	***S. mutans* UA159 (%)**	***S. pneumoniae* TIGR4 (%)**	***S. thermophilus* LMD-9 (%)**	**Name**	**Function**
SSU0061	67	66	72	CinA	DNA binding and homologous recombination
SSU0062	86	84	87	RecA	
SSU0126	66	63	58	ComY	Pilus assembly
SSU0127	58	63	53	ComYB	
SSU0128	59	65	60	ComYC	
SSU0129	48	45	44	ComYD	
SSU0130	49	42	48	ComYE	
SSU0131	47	52	47	ComYF	
SSU0132	36	37	35	ComYG	
SSU0133	60	62	61	ComYH	
SSU0144	76	75	72	SsbB	SSDNA binding and protection
SSU0610	46	46	60	ComEA	dsDNA receptor and channel
SSU0611	57	53	50	ComEC	
SSU1083	50	52	46	CoiA	Implicated in DNA homologous recombination
SSU0924	66	64	61	RadC	DNA binding and protection
SSU0393	58	66	53	ComFA	ssDNA binding and intracellular translocation
SSU0394	51	49	44	ComFC	

The *S. suis* CIN-box regulon contains a dedicated set of genes, the *comYA*-*YH* operon (*comGA*-*GH* in *S. pneumoniae*), with homology to the Type 4 pili (T4P) of Gram-positive bacteria, predicted to encode a putative ATPase (ComYA), a membrane protein (ComYB) and five other proteins corresponding to the major pilin (ComYC) and to the minor pilins (ComYD, ComYE, ComYF, ComYG, and ComYH; Laurenceau et al., 2013). With the exception of *recA*, that has its own promoter and is expressed constitutively, the *S. suis* CIN-box regulated genes were all highly expressed at 5 min and thereafter showed decreased expression, eventually returning to basal levels after 45 min. This pattern of temporal expression has also been described in *S. pneumoniae* with the difference that the ComX regulated genes of *S. pneumoniae* peak at 15 min after induction of competence rather than 5 min as we observed in *S. suis* (see clusters 3 and 4, Figure 1).

### Deletion of the major pilin gene *comYC* prevents DNA transformation

To verify if the conserved ComY operon was necessary for competence in *S. suis* we generated a *S. suis* deletion mutant of the pilin *comYC* and verified the mutation by PCR and sequencing (Table 1). The *comYC* deletion in *S. suis* prevented subsequent attempts to obtain DNA transformation after competence induction with the SigX-inducing-peptide (XIP) providing further evidence for the conserved role of ComYC in competence for DNA transformation.

### Regulation and function of DNA processing and recombination enzymes in competence

In *S. pneumoniae* it is thought that once the pilus is polymerized, a channel is formed that passes through the cell wall and the capsule, allowing the exogenous DNA to be internalized into the cytoplasm (Petersen et al., 2005; Laurenceau et al., 2015). Before or concomitant with DNA translocation, the activity of EndA generates a single stranded DNA (ssDNA) molecule. Additionally, CoiA, DprA, and RecA, a DNA-dependent ATPase, promote formation of the recombination synapse, heteroduplex formation and strand exchange between homologous DNAs (Desai and Morrison, 2006; Morrison et al., 2007).

In *S. suis* genes *coiA, radC, recA*, and *cinA* which are involved in the formation of the recombination synapse, heteroduplex formation and strand exchange were regulated by ComX (Table 2). In contrast to *S. pneumoniae* and *S. mutans*, EndA, a DNA specific nuclease that converts the dsDNA bound by ComEA and ComEC (Lacks et al., 1974; Mirouze et al., 2013) into ssDNA before or concomitant with delivery into the cytoplasm through ComEC (Seitz et al., 2014), and SsbA, single stranded binding protein A, which protects the transforming ssDNA from degradation were not regulated by ComX (Attaiech et al., 2011).

**Figure 3:**
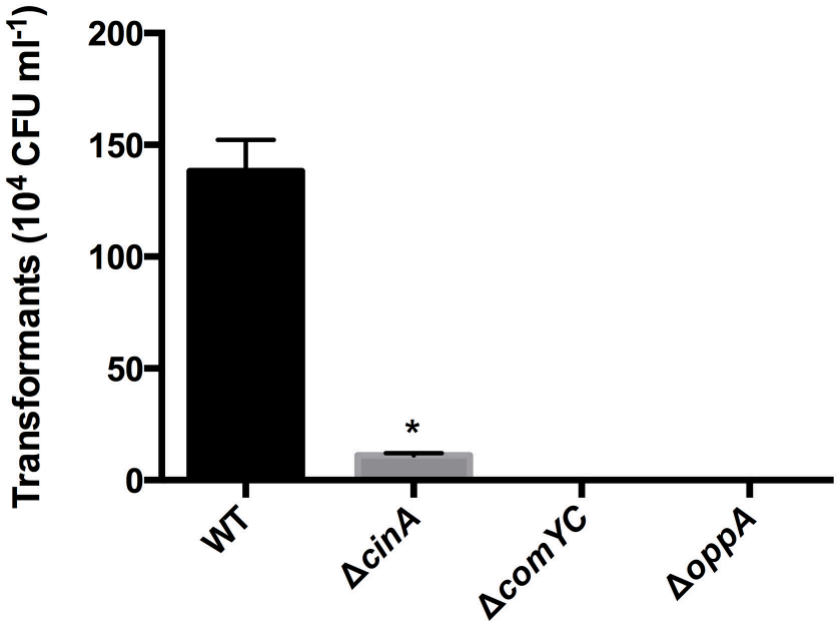
**Number of transformants obtained with Δ***cinA***, Δ***comYC***, and Δ***oppA*** deletion mutants in ***S. suis*** strain S10**. The mutants described above and in Table 1 were made using Gene Splicing Overlap Extension PCR (SOE-PCR) and allelic replacement as previously described (Zaccaria et al., 2014). The efficiency of transformation was determined by plating and enumeration of chloramphenicol resistant colonies after competence induction and transformation with plasmid pNZ8084. ^*^Indicates a significant reduction in transformation efficiency.

Pneumococcus *endA* deletion mutants were found to accumulate DNA at the cell surface (Lacks et al., 1974). There is probably a similar role for EndA in competence development in *S. suis* but we were unable to verify this as we were not able to obtain *endA* gene deletion mutants suggesting EndA has an additional essential role in *S. suis*. This is consistent with an absence of a CIN-box in the *endA* promoter and the constitutive expression of *endA* in *S. suis*. Expression of *cinA* and the downstream gene *recA*, is strongly enhanced at 5 and 15 min after XIP exposure. Unlike *cinA, recA* was constitutively expressed at a lower basal level in the absence of XIP, reflecting its “housekeeping” role in DNA recombination. To determine whether the predicted *S. suis* ortholog of CinA may have a role in competence, a *cinA* deletion mutant (Δ*cinA*) was generated using previously described methods (Zaccaria et al., 2014) and verified by PCR and sequencing. The Δ*cinA* mutant resulted in substantially reduced DNA transformation efficiency compared to the parent wild-type (WT) strain S10 (about 8% of WT efficiency) suggesting that CinA has an important but not essential role in DNA transformation (Figure 3) as shown for other bacteria (Masure et al., 1998; Mair et al., 2012).

### Expression profiles of the transcriptional regulators of competence, *comR* and *comX*

In all the streptococcal species in which natural competence has been demonstrated, its activation leads to expression of the alternative sigma factor X (*comX* or *sigX*). We have previously shown that *comX* is essential for natural transformation in *S. suis* S10 (Zaccaria et al., 2014). In our transcriptome data *comX* expression was strongly up-regulated at 5 min (1300-fold compared with the control), relatively mildly up-regulated at 15 min (105-fold) and up-regulated again at 45 min (327-fold). This fluctuation in *comX* expression could be due to an oscillation of the positive and negative feedback loops controlling its transcription (Haustenne et al., 2015).

Despite the high amount of *comX* expression at the 45 min time point, the CIN-box genes under its direct regulation were not increased in expression at 45 min compared to 15 min in the XIP-induced samples with DNA provided exogenously, although they were expressed significantly higher compared to uninduced control samples.

We have shown that ComR is required for competence induction in *S. suis* (Zaccaria et al., 2014) and postulated that ComR interacts with the mature ComS pheromone to induce *comX* and *comS*, in a similar way as described for other streptococci possessing the ComRS regulatory system (**Figure 5**) (Mashburn-Warren et al., 2010; Gardan et al., 2013). In support of this hypothesis, the expression of *comS* is similar to *comX* expression, characterized by a strong induction at 5 min, a decline at 15 min and an induction at 45 min. The *comR* regulator is constitutively expressed in broth cultures and slightly increased 5 min after peptide-induced competence (2.3-fold compared to the control) but then rapidly decreases to its prior expression level at 15 min. Microarray expression values for *comX* in the uninduced state are close to zero, preventing *S. suis* from entering the competence state, whereas in the uninduced state *comS* the competence inducing peptide precursor was expressed at low levels (Figure 1).

### Where is the exit?—regulation of competence shut down

In *S. suis* expression of MecA, the adapter protein regulating ClpCP-mediated degradation of ComX in *S. thermophilus*, was not substantially different over the time course of competence induction. *mecA* expression was down-regulated at 5 min (0.42-fold) and 45 min (0.88-fold) and up-regulated at 15 min (1.28-fold). Similar fold changes in the expression were measured for ClpC at the same respective time points. These findings make it unlikely that only these two proteins are responsible for degrading ComX and exiting the competence state. One other candidate gene that may regulate competence exit is the *dprA* gene that is regulated by ComX and has a dual role in the natural transformation system of *S. pneumoniae*. In the later species DprA promotes the homologous recombination facilitating RecA binding to the ssDNA but can also bind to the phosphorylated form of the ComE response regulator (ComE~P), preventing its interaction with the ComX promoter (Mirouze et al., 2013; Weng et al., 2013), thereby shutting down competence. The *S. suis dprA* possesses a CIN-box in its promoter and was highly induced 5 min after competence induction. However, it seems unlikely to have a role in competence shutdown because *S. suis* utilizes ComRS rather than the two-component system ComCDE as a regulatory switch for *comX* expression. *S. thermophilus*, which also uses ComRS as the proximal switch for *comX* expression does not appear to utilize DprA for shutting down *comX* expression.

### Conserved function of the oligopeptide permease gene cluster of *S. suis* in competence induction

In all investigated ComRS systems efficient binding of ComR to its operator motif is strictly dependent on the presence of the XIP pheromone (Fleuchot et al., 2011, 2013; Fontaine et al., 2013; Aggarwal et al., 2015). In other streptococci, XIP mediates the quorum sensing mechanism of competence induction by its transport back into the cell via the Opp oligopeptide ABC type transporter (Gardan et al., 2009; Mashburn-Warren et al., 2010). In *S. suis* the five genes encoding the Opp transporter are organized in two transcriptional units, an operon of four genes and OppA which encodes the subunit A of the Opp transporter complex. The expression of the full transporter system did not significantly change during competence induction. We generated a knockout of *oppA*, the component of the transporter that recognizes XIP, and were unable to transform this mutant. Although this is consistent with *oppA* deletion in other streptococcal species, we cannot completely rule out that the slower growth of the OppA mutant impacted on competence development.

### *S. suis* contains a CIN-box-regulated homolog of fratricin and a putative bacteriocin-producing operon

**Figure 4:**
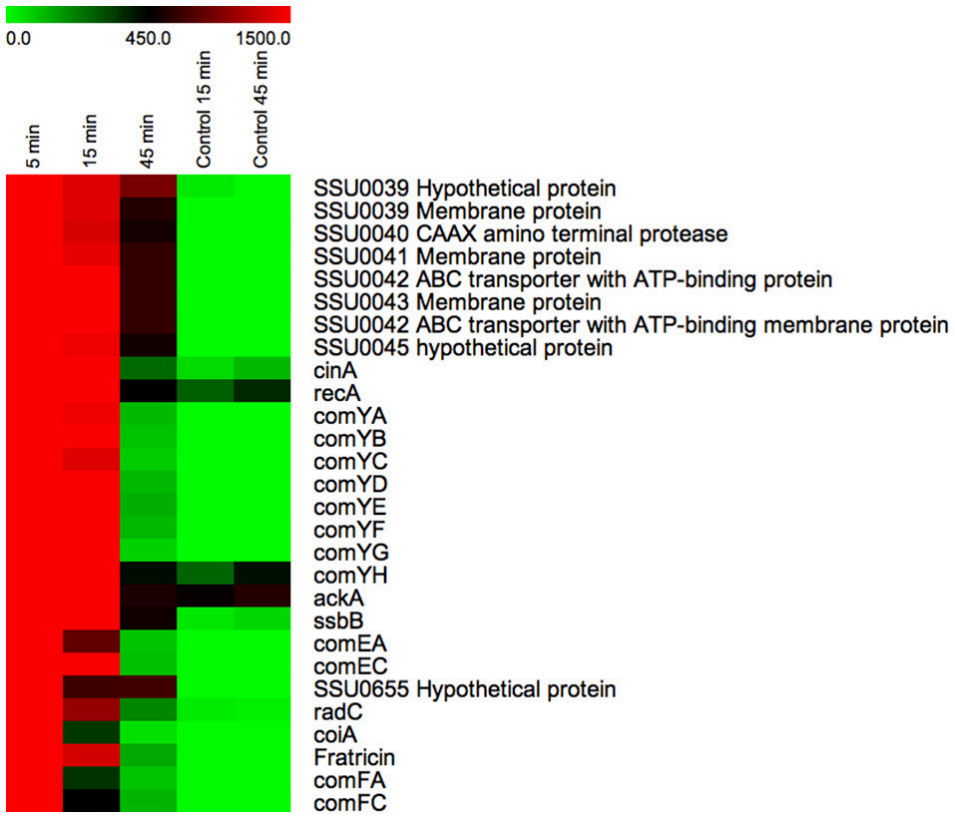
**Temporal expression of the genes under direct regulation of ComX**. Heatmap displaying the temporal expression of predicted ComX-regulated genes during competence development. The color scale at the top depicts the normalized, unlogged expression values of the genes indicated on the right. The heatmap colors represent gene expression levels from the lowest value (zero, light green) to the highest level (~3000, bright red). To not lose resolution of intermediary expression levels, a highest cut-off value of 1500 was applied to the heatmap display. The time at which bacterial samples were rapidly centrifuged and suspended in RNAprotect buffer is indicated above each column. “Control” indicates bacterial cultures to which no inducing peptide was added.

Downstream of a CIN-box promoter we identified a fratricin-like gene in *S. suis* (SSU1911) that contains an N-terminal CHAP (Cysteine, Histidine-dependent Amidohydrolases/Peptidases) domain and two SH3b (central Src homology 3b) domains, which are also present in pneumococcal fratricin (Berg et al., 2012). After induction of *S. suis* competence by XIP, a gene encoding a homolog of fratricin was up-regulated at 5, 15, and 45 min by 722-, 208-, and 22-fold, respectively. The expression profile of this gene was similar to the expression profile of the ComX-regulated genes of *S. suis* (Figure 4) suggesting that *S. suis* also produces a fratricin-like protein during competence development. *S. pneumoniae* fratricin, a cell wall hydrolase, was shown to lyse non-competent pneumococci and closely related bacterial species, thereby ensuring that the transforming DNA has overall a high level of homology to the competent recipients, favoring beneficial DNA recombination over detrimental genetic events (Kausmally et al., 2005; Håvarstein et al., 2006; Claverys et al., 2007; Berg et al., 2012). We speculate that the *S. suis* fratricin-like protein may have a similar role to that of pneumococcal fratricin, although we could not identify a homolog of the candidate fratricin immunity gene ComM, described in *S. pneumoniae* (Håvarstein et al., 2006; Eldholm et al., 2010).

Interestingly, we also identified a CIN-box regulated operon consisting of eight genes that do not show significant homology with other competence genes. The operon SSU0038-45, comprises three putative membrane proteins, one CAAX amino terminal protease and two ABC transporters with ATPase activity. In addition, we measured high expression of two relatively small putative ORFs (SSU0038 and SSU0045) represented on the microarray but not annotated in *S. suis* genome. These ORFs are predicted to encode two small (42 and 57 amino acids) proteins with unknown function but their size and association with a CAAX peptidase and two ABC transporters suggests a possible role as bacteriocins. This is also supported by the peptide leader sequence of SSU0045 that features a double-glycine motif which is a characteristic of bacteriocins that are secreted by ABC transporters (van Belkum et al., 1997). In *S. mutans*, competence induction directly controls bacteriocin production (Reck et al., 2015). Also in *S. gordonii*, a locus with a CIN-box in the promoter region that encodes a bacteriocin has been reported. The competence-related bacteriocin peptide in *S. gordonii* also contains a double-glycine motif for export via an ABC-type transport system (Håvarstein et al., 1995) and is active against *S. gordonii* and *S. mitis* (Heng et al., 2007).

## Concluding remarks

**Figure 5:**
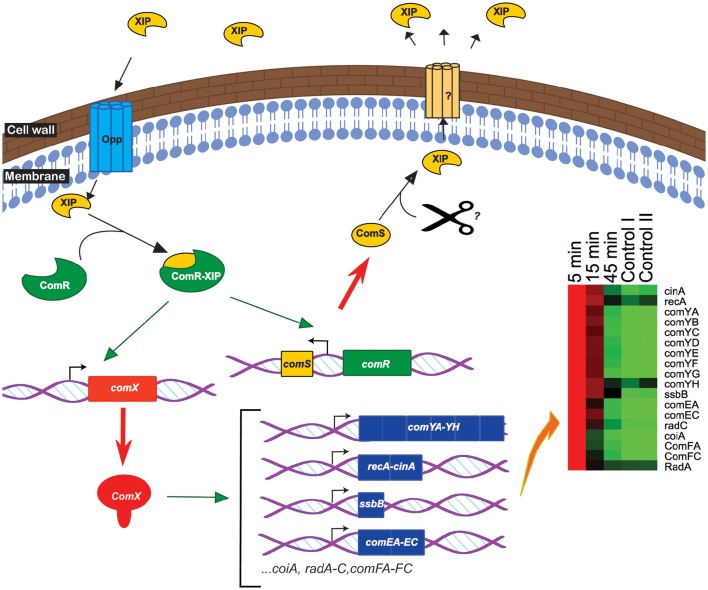
**Simplified model of competence induction in ***S. suis*****. Extracellular SigX-inducing-peptide (XIP) enters the bacteria via the Opp transporter system. Intracellularly, the transcriptional regulator ComR binds to XIP and the ComR-XIP complex promotes the expression of *comS*, encoding the full-length form of the XIP pheromone, and of *comX* (green arrow). ComX activates the expression (green arrow) of the late-competence genes involved in the transformasome having a CIN-box in their promoter (heatmap). ComS is processed and secreted by an unknown mechanism inducing a positive feedback loop.

As far as we are aware this is the first transcriptomics study of the complete time course of competence development in a streptococcal species harboring a ComRS system. This time-resolved overview of the genetic regulation of competence revealed the ComX regulon, comprising all genes encoding homologs of all the known transformasome proteins in *S. pneumoniae, S. mutans*, and *S. thermophilus* (Figure 5, Table 2). Additionally, we showed that deletion mutants of the major pilin gene *comYC*, which is required for formation of the DNA binding pilus is necessary for peptide-induced DNA transformation. A mutant of *cinA*, encoding a protein involved in DNA binding and recombination was strongly attenuated for DNA transformation. *OppA* encoding the binding subunit of the general oligopeptide transporter was required for competence development suggesting it transports XIP into the bacteria where it binds to ComR. In contrast to previous studies with *S. pneumoniae*, endA, encoding a DNA specific nuclease that converts the dsDNA bound by ComEA and ComEC into ssDNA during uptake by the transformasome (Lacks et al., 1974; Mirouze et al., 2013) could not be deleted in *S. suis* suggesting it might have an additional essential role in this species. *S. suis* expresses a fratricin-like gene and a putative bacteriocin and associated transport system during competence development, which we speculate may play roles in acquiring DNA as described for other species. The induction of competence was transient with expression of the ComX-regulated genes peaking at around 5 min after addition of the peptide and declining substantially at 15 min, despite continued presence of *comX* transcripts to 45 min. The transient nature of competence development is assumed to avoid potentially adverse effects of genetic recombination on genome integrity during cell division and is associated with a suppression of basal metabolism (Zaccaria et al., 2016b). From the transcriptomics data alone it was not possible to identify genes regulating exit from competence and further studies are needed to elucidate the mechanisms involved.

## Author contributions

JW, EZ, Pv, and MW contributed substantially to aspects of the design of the experimental work and the interpretation of data. EZ, JW, and Pv contributed to the conception of the whole study. EZ performed the experimental work. MW, Pv, and EZ were involved in the acquisition and analysis of data. JW and EZ wrote the draft manuscript. Pv, MW, EZ, and JW revised the intellectual content of the manuscript, and approved the final version for submission. All authors agree to be accountable for all aspects of the work in ensuring that questions related to the accuracy or integrity of any part of the work are appropriately investigated and resolved.

## Funding

This work was supported by the European Commission, as part of the Framework Programme 7, Marie Curie Initial Training Network - STARS (Contract No. PITN-GA-2009-238490).

### Conflict of interest statement

The authors declare that the research was conducted in the absence of any commercial or financial relationships that could be construed as a potential conflict of interest.
